# Post-traumatic massive hand lymphedema fully cured by vascularized lymph node flap transfer

**DOI:** 10.1051/sicotj/2018049

**Published:** 2018-11-27

**Authors:** Corinne Becker, Lionel Arrivé, Giuseppe Mangiameli, Ciprian Pricopi, Fanomezantsoa Randrianambinina, Francoise Le Pimpec-Barthes

**Affiliations:** 1 Department of Thoracic Surgery and Lung Transplantation, Paris Descartes University, Hôpital Européen Georges Pompidou, Assistance Publique - Hôpitaux de Paris, Paris France; 2 Department of Radiology, Sorbonne Universités, UPMC University Paris 6, Saint Antoine Hospital, Assistance Publique − Hôpitaux de Paris, Paris France; 3 INSERM UMR-S 1162, Paris France

**Keywords:** Hand traumatism, Lymphedema, Magnetic resonance lymphography, Lymph node flap transfer, Dermolipectomy.

## Abstract

Post-traumatic localized hand lymphedema is a rare situation and its diagnosis may be difficult, causing lack of care leading to failure of care. Our case study is of two young women with massive post-traumatic hand lymphedema who were treated for algodystrophy for 2 years, and whose bandages and physiotherapy were unsuccessful. Major social and psychological consequences due to difficulty with diagnosis and management resulting in inappropriate tests and therapeutic treatment were prescribed due to these issues. Noncontrast magnetic resonance lymphography revealed complete lymphatic vessel blockage in the hand and wrist. A vascularized lymph node flap harvested at the groin level was transferred to the elbow level 1 month after local dermolipectomy. These procedures resulted in the restoration of lymphatic flow. Both patients were definitely cured, and they returned to normal life within 6 months after surgery. Lymph node flap transfer associated with dermolipectomy may cure massive localized lymphedema in selected cases.

## Introduction

Lymphedema of the arm as a complication of surgery for breast cancer is the most common situation and its etiologies are well known. However, many physiological questions persist concerning spontaneous, congenital, or post-traumatic forms of this disease. To our knowledge, no series occur in literature concerning post-traumatic and post-burn lymphedema. Only rare cases describe lymphedema of the hand following a fracture of the distal radius, and the role that the traumatism played was controversial [1]. Older data exit, reporting periods of hand lymphedema coinciding with flares of rheumatoid polyarthritis [2]. In such cases, lymphoscintigraphy has indicated lymphatic ectasia or obstruction [2]. The improving quality of magnetic resonance imaging (MRI) allows for the detection of lymphatic vessel (LV) malformations. In fact, lymphedema can be misdiagnosed for long periods of time and therefore inappropriately treated. However, 40% of (stages 1 and 2) patients can be cured by vascularized lymph node flap transfer (VLNFT) and the others experience improvement [3]. This flap promotes the spontaneous growth of the lymphatic vessels, allowing the lymph to join the venous circulation [3]. Our case histories are two post-traumatic cases of localized hand lymphedema, upon which delayed diagnosis and ineffective therapy was of disastrous consequences on hand function and the patients' psychology, social insertion, and general prospects of life. The two patients agreed and even insisted on the necessity of publishing their exceptional and difficult medical course histories.

## Case 1

**Figure 1:**
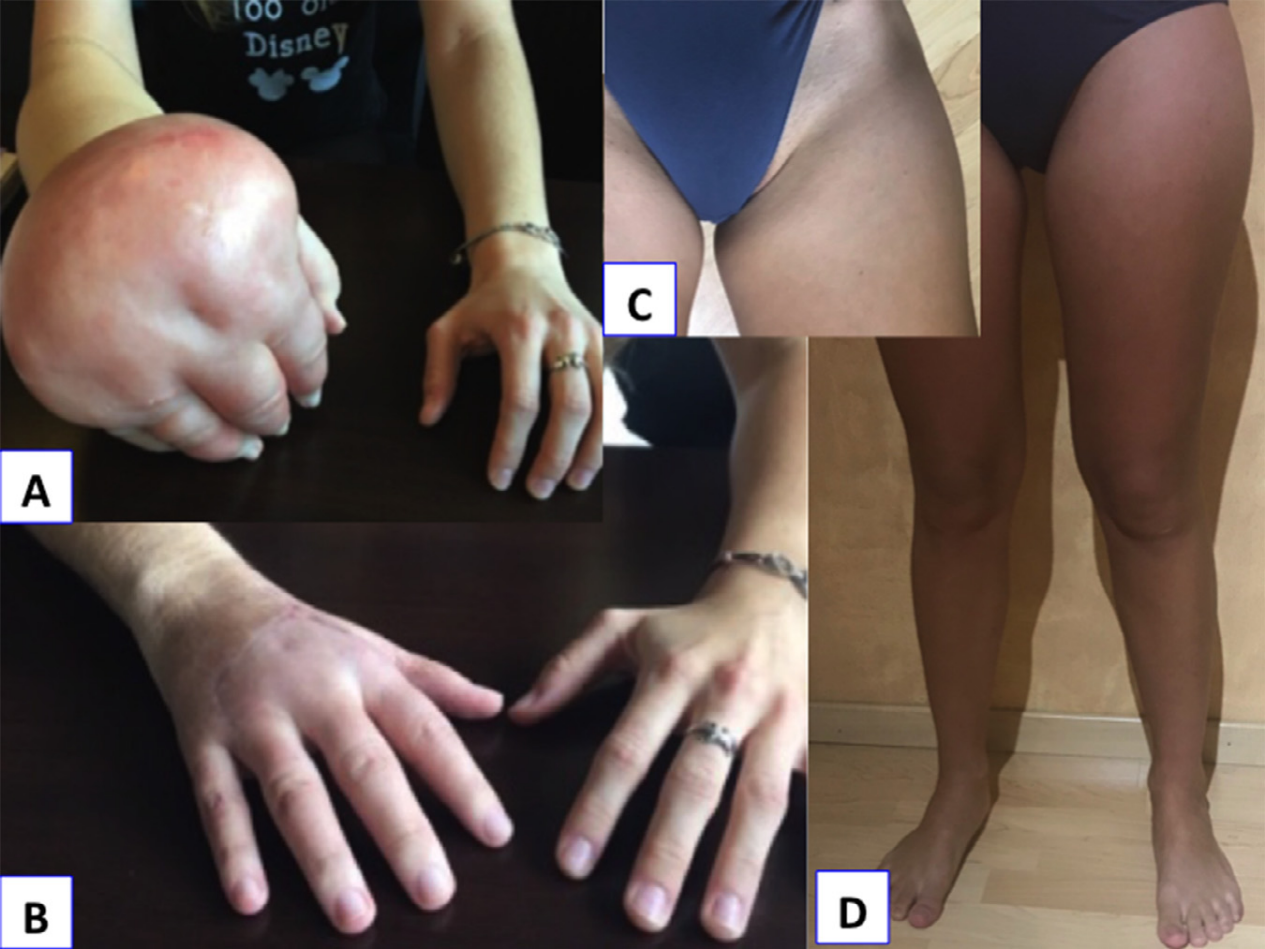
Case No. 1. (a) Clinical examination in the 21-year-old woman before surgery demonstrating a huge hand lymphedema. (b) Normal right hand 6 months after surgery. (c) Scar at the donor site below the iliac crest 1 year after surgery. (d) No lymphedema exists in the lower limbs 1 year after the lymph node flap harvesting.

**Figure 2:**
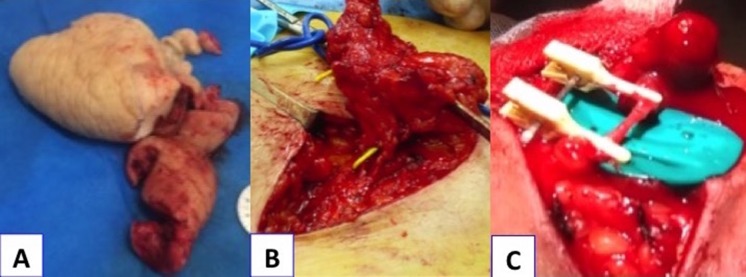
Peroperative procedure. (a) Product of extrafascial dermolipectomy at hand level. (b) The flap containing the nodes around the superficial vein is elevated deeply onto the fascia, to the superficial iliac artery and the veins that emerge from the femoral vein and artery, at the internal part, near the pubis crest. (c) The flap placed in the elbow region. Microsurgical anastomosis is performed on the microscope, with separated points (6–8) on the receiving vessels (Nylon 10 × 0).

A 21-year-old woman without former medical history had a bicycle accident resulting in her right-hand traumatism. No fractures were detected on X-rays. Wrist sprain was suspected because of persistent swelling. A plaster cast was applied for 15 days. Physiotherapy, including manual drainage, was performed. Two months later, the hand was still inflated and painful. Algodystrophy was suspected following a bone scan. Venous Doppler ultrasound of the upper limb excluded the diagnosis of phlebitis. The hand was so swollen (Figure 1a) and painful that the young woman stopped her studies. Massage, manual drainage, and intravenous biphosphonate treatment were delivered without improvement. The patient was suspected of self-injury with strangling threads at wrist level. MRI angiography ruled out arteriovenous malformation. Two series of intravenous immunoglobulin were delivered without improvement. The final proposed diagnosis was a psychiatric disease corresponding to Munchausen syndrome or Secretan's syndrome, a possible etiology of hand lymphedema. Personality tests and psychiatric evaluations were done but the results were negative. A total impairment of the hand complicated by recurrent local infection appeared. The rheumatologists organized a consultation with specialists in lymphology. The first specialist decided to continue the physiotherapy treatment. The patient asked for a second opinion from in our center. The noncontrast magnetic resonance lymphography (NCMRL) demonstrated local lymph flow (LF) blockage at the wrist. It was associated with lymphatic malformations of the entire forearm, justifying a surgical procedure. An extrafascial dermolipectomy was first performed (Figure 2a). Local advanced flaps were performed to close the hand. This first step was essential to remove all fibrous and nonfunctional tissue obstructing lymphatic circulation. This allowed for the removal of the strangulation at the wrist. One month following dermolipectomy, the time necessary for the hand to heal later, a free flap containing some lymph nodes VLNFT was performed. The autologous donor flap harvested at groin level (Figure 2b) was transferred to elbow level. It was supplied by superficial circumflex iliac artery and contained lymph nodes, lymphatic vessels, and fat. One arterial and one venous anastomosis were performed to connect flap vessels to perforate branches (artery and vein) around the elbow (Figure 2c). Lymphatic vessels were kept intact, allowing the spontaneous anastomoses to appear, which are the signs of lymphatic growth. The postoperative course was uneventful with rapid functional recovery. At the 1-year follow-up, the hand was nearly normal (Figure 1b) and there is no lower limb lymphedema (Figures 1c and 1d). The patient returned to the university and could play the piano and the guitar again.

## Case 2

**Figure 3:**
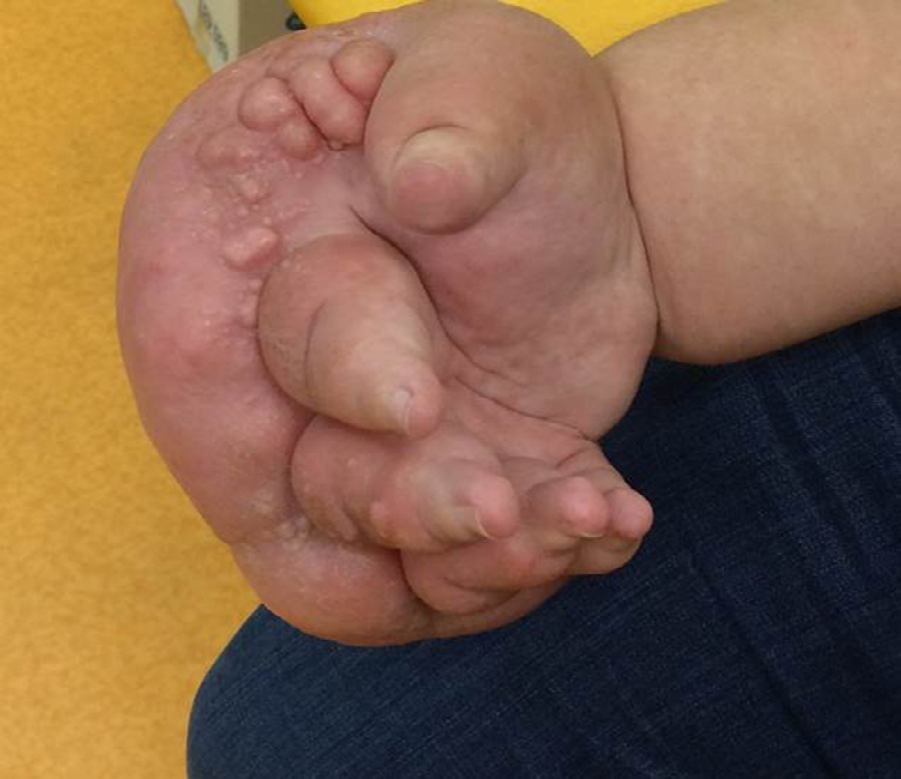
Clinical examination in the 26-year-old woman (Case No. 2) before surgery, demonstrating a huge hand lymphedema.

**Figure 4:**
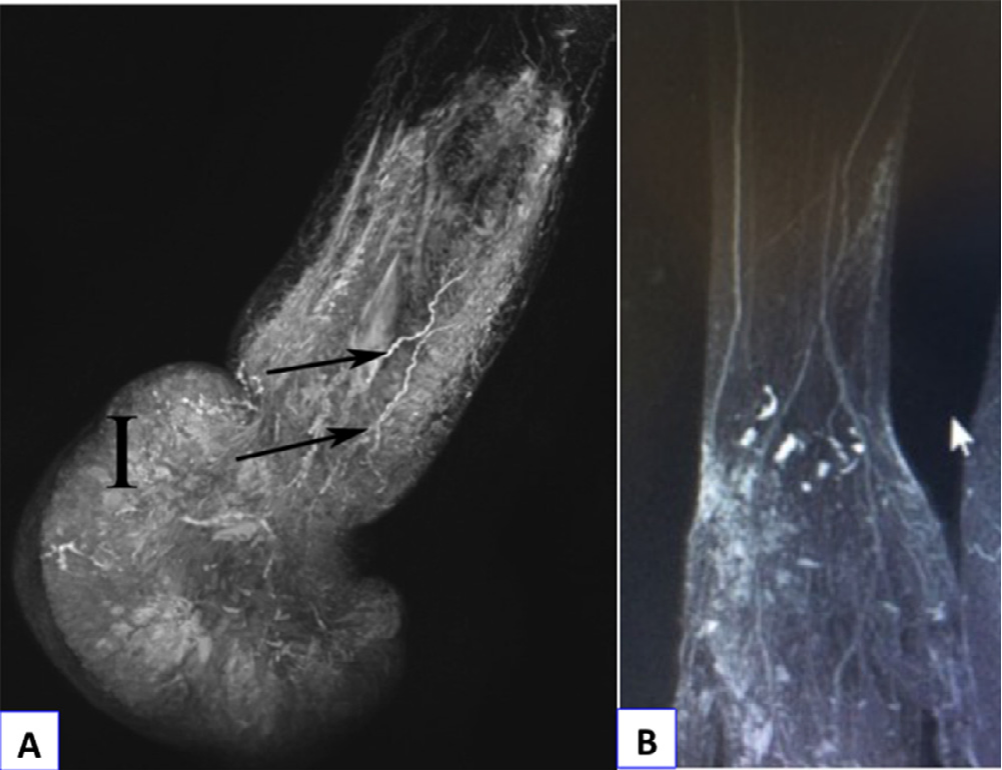
(a) Noncontrast magnetic resonance lymphography before surgery in the 26-year-old woman (Case No. 2) demonstrating lymphedema of right hand with subcutaneous tissue infiltration (I) and dilatation (arrows) of distal lymphatic vessels of the forearm. (b) Normal noncontrast magnetic resonance lymphography of the wrist and forearm.

**Figure 5:**
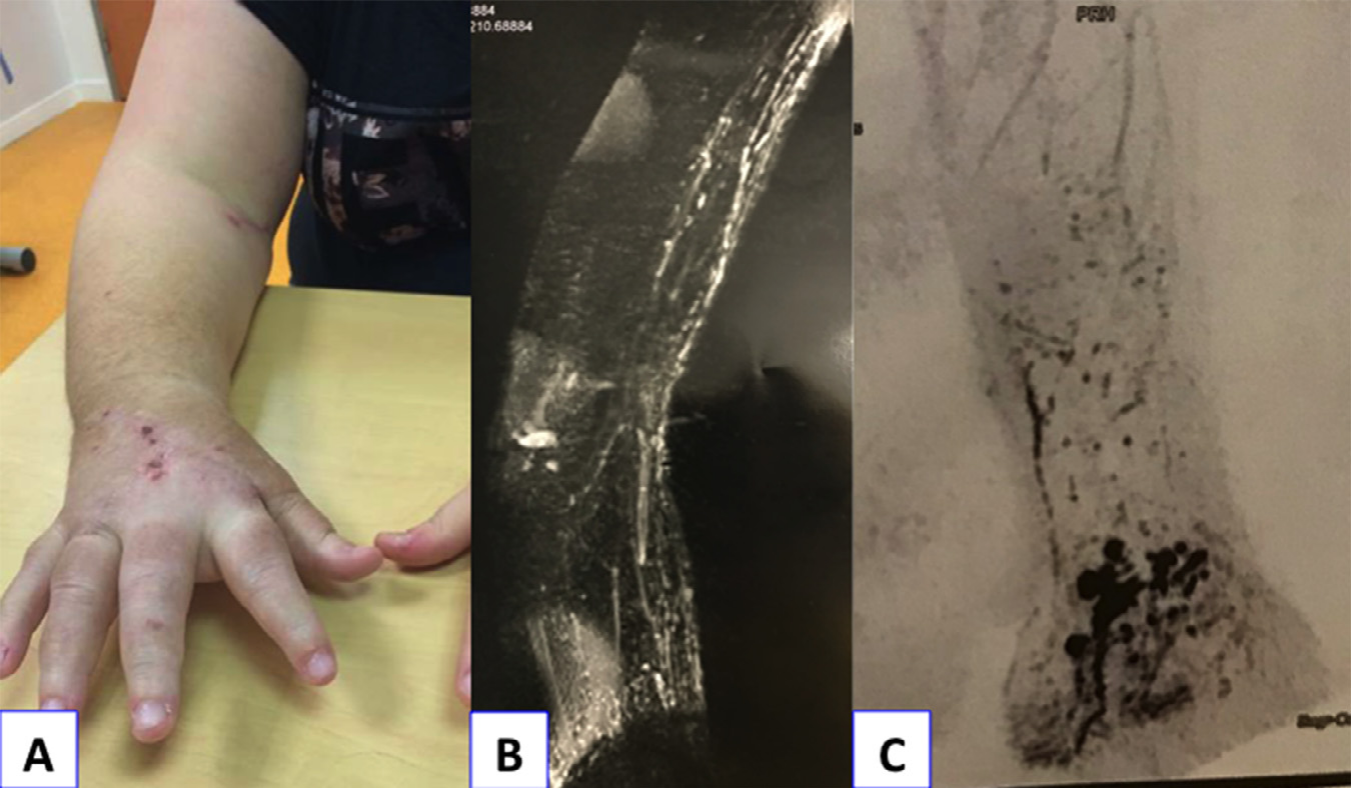
Case No. 2 after surgery. (a) Clinical examination in the 26-year-old woman (Case 2) 6 months after surgery, showing a normal right hand. (b) Noncontrast magnetic resonance lymphography 1 year after surgery showing new lymphatic vessels at elbow level. (c) Noncontrast magnetic resonance lymphography 1 year after surgery showing normal lymphatic circulation at wrist and hand level.

A 26-year-old woman without former medical history had had a right-hand lymphedema for 4 years, which evolved quickly within a few weeks. The origin was apparently a burn of the hand. Six months after analgesic treatment, the patient was referred to a lymphology center. She was told that it was a chronic disease without any possibility of recovery and could be treated by massages, lymphatic drainage, and bandages. Unable to work because of hand impairment, she lost her job and could not find a new job because of the monstrous aspect of her hand (Figure 3). Her referring physician could not find any solution and the patient was referred to a pain treatment center. At that time, the hand weighed 6 kg. The lymphedema was painful and required daily morphine intake. Via social networks, the young woman found a “lymphedema group” that mentioned treatment by VLNFT, so she came to our center. An obstruction of the LF at elbow level was confirmed by NCMRL (Figure 4a) compared to normal aspect (Figure 4b). A two-stage surgical procedure was decided consisting of an extrafascial dermolipectomy of the hand, followed, 1 month later, by a VLNFT from the groin area to the elbow. The same surgical procedure as described in case 1 was performed. In the postoperative course, forearm compression by bandage was maintained. The hand quickly ameliorated (Figure 5a) and was functional after 6 months, allowing the patient to work again. The postoperative NCMRL showed new LVs at elbow level (Figure 5b) and normal LF (Figure 5c).

## Discussion

Post-traumatic hand lymphedema is a rare lymphatic disease and is most certainly due to a local dysplasia of a weak lymphatic system, deteriorated by infections. In our two case studies, the diagnosis was so delayed that the prognosis worsened considerably. Despite new recently published treatments [1], adapted treatments for limb lymphedema, remain marginal and not well codified. The delay in lymphedema diagnosis and therapy was reported as over 2 years by Ozturk et al. [4], who reviewed 18 studies collecting 305 patients. Massages and lymphatic drainage are usually indicated to improve the LF and diminish lymph-stasis in the limbs but they are insufficient in major LVs interruptions. Our two cases illustrate the difficulty in establishing a correct diagnosis and show the isolation of patients who are not offered curative surgical treatments.

Hand lymphedema mechanisms are poorly defined. An external factor (skin burn, direct trauma) may decompensate a congenital malformation by sudden or progressive blockage of the LF in malformed LVs. At wrist level, the diffusion spaces are restricted and the phenomena can quickly lead to important complications as can be observed in carpal tunnel syndrome post-lymphedema. High tissue pressure might also block LVs regeneration usually occurring after LV injury. Misdiagnosing the disease as an incurable chronic illness might have had irreversible consequences (hand amputation, suicide). For our two patients, NCMRL and surgery fortunately helped avoid these fatal outcomes.

Concerning lymphatic imaging, bipedal-lymphography is not suitable and lymphoscintigraphy is not always appropriately suitable. In fact, this exam is of no interest in the case of obliterated LVs, because it requires functional LF. Nowadays, NCMRL enables the exploration of complex lymphatic abnormalities [5] and provides complete thoracic and abdominal views. It may help clarify the diagnosis and guide the patient to the right specialist. Because we now consider that NCMRL is essential in finding out the best treatment, we use it as soon as complex chylothorax or limb lymphedema is suspected.

The surgical technique of VLNFT is based on two essential notions: LVs anatomy and knowledge of LVs growth patterns − lymphangiogenesis. Indeed, VLNFT brings vascular endothelial growth factor-C-producing tissue, favoring local lymphangiogenesis and bridging the distal system with the proximal lymphatic system. Harvesting lymph nodes (LNs) with their feeding blood vessels and transferring them to another site without disrupting the donor-site LF [3] is safe as long as anatomic knowledge is respected. The dissection begins externally. The flap containing the nodes around the superficial vein is elevated deeply onto the fascia, to the superficial iliac artery and the veins that emerge from the femoral vein and artery, at the internal part, near the pubis crest (Figure 2b). This flap can be enlarged in the medial part of the suprapubic region, and in the lateral part of the iliac crest (mac gregor flap). This flap containing lymph nodes can include fat and skin because of the circumflex iliac vessels. The inguinal ligament is the lower margin of the flap and remains superficial (it does not harvest deep inguinal nodes). This flap is placed in the elbow region. Microsurgical anastomosis is performed on the microscope, with separated points (6–8) on the receiving vessels (Nylon 10 × 0) (Figure 2c). The diameter of the vessels is around 1 mm.

Secondary lymphedema due to donor-site LV injury is rare and its severity is never comparable to that of the treated lymphedema. The analysis of LVs anatomy by NCMRL allows verification of LNs transfer feasibility and prevention of such complications [6] − in the case of malformation or LVs scarcity, another donor site can be found.

To conclude, we demonstrated that lymph node flap transfer associated with dermolipectomy may cure massive localized lymphedema in selected cases. However, to date, this technique has been performed only by a few expert surgeons who remain the sole references for this atypical pathology.

## Conflict of interest

The authors declare that they have no conflicts of interest in relation to this article.
